# The tumor-stroma ratio in giant cell tumor of bone: associations with the immune microenvironment and responsiveness to denosumab treatment

**DOI:** 10.1186/s13018-024-04885-8

**Published:** 2024-07-15

**Authors:** Hai-Lin Wu, Xiao-Bin Wang, Jing Li, Bo-Wen Zheng

**Affiliations:** 1 Department of Spine Surgery, The Second Xiangya Hospital, Central South University, 139 Renmin Road, Changsha, 410011 Hunan China; 2 Musculoskeletal Tumor Center, Peking University People’s Hospital, Peking University, No. 11, Xizhimen South Street, Xicheng District, Beijing, 100044 China

**Keywords:** Tumor-stroma ratio, The immune microenvironment, Prognostic biomarker, Denosumab, Giant cell tumor of bone

## Abstract

**Background:**

Currently, there is limited understanding regarding the clinical significance of the tumor-stroma ratio (TSR) in giant cell tumor of bone (GCTB). Hence, we aimed to investigate the distribution of TSR in GCTB and explore its correlation with various clinicopathologic factors, immune microenvironment, survival prognosis, and denosumab treatment responsiveness.

**Methods:**

We conducted a multicenter cohort study comprising 426 GCTB patients treated at four centers. TSR was evaluated on hematoxylin and eosin-stained and immunofluorescent sections of tumor specimens. Immunohistochemistry was performed to assess CD3+, CD4+, CD8+, CD20+, PD-1+, PD-L1+, and FoxP3+ TIL subtypes as well as Ki-67 expression levels in 426 tissue specimens. These parameters were then analyzed for their correlations with patient outcomes [local recurrence-free survival (LRFS) and overall survival (OS)], clinicopathological features, and denosumab treatment responsiveness.

**Results:**

Low TSR was significantly associated with poor LRFS and OS in both cohorts. Furthermore, TSR was also correlated with multiple clinicopathological features, TIL subtype expression, and denosumab treatment responsiveness. TSR demonstrated similar predictive capabilities as the conventional Campanacci staging system for predicting patients' LRFS and OS.

**Conclusion:**

The results of this study provide evidence supporting the use of TSR as a reliable prognostic tool in GCTB and as a predictor of denosumab treatment responsiveness. These findings may aid in developing individualized treatment strategies for GCTB patients in the future.

**Supplementary Information:**

The online version contains supplementary material available at 10.1186/s13018-024-04885-8.

## Introduction

Giant cell tumor of bone (GCTB) is a malignant bone tumor that exhibits osteolytic behavior and has a recurrence and metastasis rate of approximately 27–65% [1]. Despite the fact that surgical curettage in conjunction with local adjuvant therapies, such as phenol, alcohol, or liquid nitrogen, can yield a positive therapeutic outcome, the postoperative local recurrence rate remains alarmingly high, ranging from 15 to 50% [2, 3]. While the administration of denosumab, a receptor activator of nuclear factor-kappa B ligand (RANKL) inhibitor, has been shown to elicit a favorable tumor response in the short term, it can also lead to an elevated risk of tumor recurrence after curettage and may even prompt GCTB to evolve into a more malignant sarcoma [4]. In light of the dismal prognosis associated with GTCB, the development of superior treatment modalities to enhance patient survival is an urgent necessity.

Currently, there exists a dearth of reliable prognostic indicators for predicting treatment response in patients with GCTB. Despite the widespread utilization of the Campanacci staging system for guiding clinical management and stratifying prognostic risk, robust predictive models remain wanting in comparison to their counterpart [5]. Although certain molecular markers have been identified as related to the survival and prognosis of GCTB, their study is restricted to the molecular level, thereby risking the dissemination of imprecise prognostic information [6, 7]. Furthermore, the stromal cells and tumor cells within the microenvironment are intricately linked [8, 9], and the tumor stroma is recognized to play a crucial role in tumor formation, progression, invasion, and metastasis [10, 11]. The expression of particular genes within the stroma has been strongly correlated with the survival and prognosis of numerous human malignancies [9, 12, 13]. In general, patients with a low tumor-stroma ratio (TSR) have a poorer prognosis [8, 9], but the relationship between TSR and clinical data among GCTB patients has yet to be explored.

The tumor immune microenvironment plays a pivotal role in tumor development, and existing literature supports the utility of immune cells within the microenvironment as prognostic markers in GCTB [6]. Recent investigations have highlighted the contentious nature of the long-term efficacy of denosumab in GCTB, albeit it remains one of the most efficacious drugs in the realm of oncology [2]. Presently, our understanding suggests that the tumor stroma impedes the immune response to tumors [14], but the connection between TSR and immune parameters, and whether patients with distinct TSRs exhibit disparate therapeutic responses to denosumab treatment, remains poorly understood. As such, the purpose of this study is to evaluate the clinical significance of TSR by examining the associations between immune microenvironment, denosumab treatment responsiveness, clinicopathological characteristics, and patient prognosis.

## Methods

### Study design

This retrospective study was conducted across multiple treatment centers, spanning a period from January 2012 to December 2021. Following exclusion criteria, a total of 426 patients diagnosed with GCTB were selected for inclusion. All patients underwent surgical treatment, and confirmation of pathological diagnosis was ensured by independent evaluation by at least two pathologists, utilizing HE biopsy and H3.3G34W results [15, 16]. Representative images of this confirmation are included in the Supplemental Digital Content. Figure S1. The patients were divided into a training cohort comprising 173 individuals from one treatment center, and a validation cohort containing 253 patients from the remaining three centers. Ethical clearance was obtained from the ethics committees of all four treatment centers, and written informed consent was obtained from each patient. The clinical cohort was thus established, as illustrated in Supplemental Digital Content. Figure S2.

### Patient and tissue samples

#### Follow-up of patients

Patient clinical data were extracted from their medical records. Campanacci stage was evaluated based on preoperative imaging [17]. The type of surgical resection was classified into wide resection (e.g., gross total or en bloc resection with negative margins) or non-wide resection (including intralesional or marginal resection or curettage) using previously established methods [18]. Moreover, denosumab was administered to 57 of the included patients after surgery, in accordance with treatment requirements [19]. The drug's effectiveness was assessed according to the Response Evaluation Criteria in Solid Tumors (RECIST) [20], and patients were classified into the ineffective group (tumor progression) and the effective group (complete remission, partial remission, stable) [21].

All included patients were those who were diagnosed with GCTB for the first time, with no instances of recurrence. All patients were monitored with regular clinical and imaging assessments. The primary outcome measures of interest were local recurrence-free survival (LRFS), defined as the time span from tumor resection to the initial local tumor recurrence as evaluated by MRI findings and/or pathologic findings on the resected specimen [21], and overall survival (OS), defined as the duration from tumor resection to all-cause mortality.

### Immunofluorescence

The Opal manual IHC kit (PerkinElmer, Waltham, Massachusetts) was utilized to perform immunofluorescence (IF), as previously described [9]. Tissues were stained with Anti-H3.3G34W antibody, which demonstrated good sensitivity and specificity for GCTB diagnosis, to generate a tumor mask. The nucleus was identified with 4′,6-diamidino-2-phenylindole (DAPI; PerkinElmer). Representative images are presented in Supplemental Digital Content. Figure S3. The tumor compartment displayed an H3.3G34W positive region, while the stroma compartment excluded the tumor mask from the DAPI compartment.

### Evaluation of the TSR

To evaluate the TSR, HE-stained tissue sections were examined based on methods previously reported in the literature [9, 22]. After the HE-stained sections and IF-stained sections were scanned and visualized using a Zeiss Axioscan 7 microscope (Carl Zeiss AG, Oberkochen, Germany), the area with the most obvious tumor infiltration was selected with a 4 × objective lens. Next, the region with the richest stroma was chosen with a 10 × objective within this region based on the heterogeneity of the tumor. Regions of the image containing tumor cells and their surrounding stromal components were used for evaluation. The assessment of results was independently conducted by two experienced pathologists who were blinded to the patients' data. In case of discrepancies, resolution was achieved through mutual consensus between the two pathologists or consultation with a third pathologist. During TSR assessment, areas with tumor necrosis and hemorrhage were excluded under direct vision, and the average value of TSR data obtained by the two methods was recorded as the final result.

### Immunochemistry

Immunohistochemical staining was conducted as previously delineated [7, 9]. In brief, 4 μm paraffin-embedded sections of 427 GCTB specimens from four institutes were deparaffinized in xylene, rehydrated using a series of graded ethanol solutions, and rinsed in distilled water. Following antigen retrieval and blocking, tissue sections were exposed to primary antibodies (Supplemental Digital Content. Table S1) overnight at 4 °C. Subsequently, immunodetection was carried out using streptavidin-peroxidase conjugate (Auragene, Changsha, Hunan, China) after incubation with a second biotinylated goat anti-rabbit or anti-mouse immunoglobulin, and then developed with 3,3-diaminobenzidine solution and counterstained with hematoxylin.

### Automated image analysis

Automated quantification of CD3, CD4, CD8, CD20, programmed cell death protein-1 (PD-1), programmed cell death-1 (PD-L1), and FoxP3 tumor-infiltrating lymphocytes (TILs) was carried out as described earlier [21, 23]. In brief, slices were examined in the TILs focal-rich area using a Zeiss inverted Eclipse Ti microscope, and representative regions of interest were photographed using the Zeiss Axioscan 7 microscopic imaging machine with Halo Visiopharm image analysis software. The number of TILs was then enumerated in 5 hot spots (20×) using a computer-assisted image analysis method. Finally, measurements were determined as the average number of positively stained cells per square millimeter (mm2). The Ki-67 expression level was computed by dividing the target marker pixel intensity by the corresponding mask's area (recorded as integrated optical density [IOD]/10^6^ pixels).

### Statistical analysis

Statistical analysis was conducted using IBM Inc.'s SPSS 26.0 software package (Armonk, New York). Categorical data were expressed as frequencies and analyzed using the chi-square test. Quantitative data were presented as mean ± standard deviation and analyzed using t-test or one-way ANOVA. The Pearson or Spearman correlation test was utilized to observe the correlation between two continuous variables. X-Tile software was used to determine cutoff values for continuous variables in survival analysis, where overall survival (OS) was the outcome parameter [24]. Patients were classified into two subgroups based on the cutoff point (≤ cutoff or > cutoff) and adjusted accordingly [25]. Univariate analysis was conducted using the Kaplan–Meier method to analyze the differences in survival between groups. Following the identification of significant covariates in univariate analysis, a multivariate Cox proportional hazards model was utilized to identify independent factors significantly associated with local recurrence-free survival (LRFS) and OS. The Bland–Altman test will be utilized to evaluate the consistency of TSR measures between the two evaluators [9]. Receiver operating characteristic (ROC) curves will be utilized to compare the predictive ability of the Campanacci staging system and TSR. All tests were two-tailed, and a *P*-value of ≤ 0.05 was regarded as statistically significant.

## Result

### Patient characteristics

Detailed characteristics of the 426 GCTB patients included in this study are presented. No differences in clinicopathological features were observed between the training and validation cohorts, with the exception of age, duration of symptoms, and CD8 + TILs, two cohorts showed good consistency, which are elaborated in Table 1. Representative images illustrating TSR are displayed in Supplemental Digital Content. Figure S4. The distribution of TILs in both cohorts is depicted in Supplemental Digital Content. Figure S5A and B. Supplementary Digital Content. Figure S6 displays representative images of high and low expression of Ki-67 and TILs. For the survival analysis, Ki-67, TILs density, and TSR in the two cohorts were categorized into low and high groups using cutoff points obtained from X-Tile software (Supplementary Digital Content. Figures S7 and S8).

**Table 1:** Comparison of baseline characteristics between training cohort and validation cohort

Factors	Categories	Training cohort (n = 173)	Validation cohort (n = 253)	*P*-value
Age (years)	Continuous	38.76 ± 16.51	32.21 ± 12.25	** < 0.001**
Gender	Female	61	99	0.476
	Male	112	154	
Tumor location	Extra-axial	117	183	0.331
	Axial	56	70	
Duration of symptoms (months)	Continuous	17.04 ± 24.71	11.06 ± 21.10	**0.010**
Preoperative neurological dysfunction	No	136	208	0.382
	Yes	37	45	
Postoperative neurological dysfunction	No	119	186	0.325
	Yes	54	67	
Tumor size (in diameter, cm)	Continuous	4.71 ± 2.83	4.97 ± 2.91	0.352
Type of resection	Wide	97	147	0.691
	Not wide	76	106	
Campanacci stage	I	16	27	0.673
	II	60	95	
	III	97	131	
Denosumab	No	153	216	0.620
	Ineffective	12	24	
	Effective	8	13	
TSR	Continuous	0.49 ± 0.27	0.49 ± 0.28	0.999
Ki-67 + expression (%)	Continuous	19.62 ± 13.93	19.17 ± 13.07	0.735
CD3 + TILs (cells/mm^2^)	Continuous	407.12 ± 317.52	407.53 ± 321.79	0.990
CD4 + TILs (cells/mm^2^)	Continuous	396.36 ± 329.02	416.12 ± 323.13	0.539
CD8 + TILs (cells/mm^2^)	Continuous	99.34 ± 117.52	190.00 ± 143.81	** < 0.001**
CD20 + TILs (cells/mm^2^)	Continuous	244.44 ± 265.82	246.27 ± 267.73	0.945
PD-1 + TILs (cells/mm^2^)	Continuous	134.55 ± 99.87	134.32 ± 102.90	0.982
PD-L1 + TILs (cells/mm^2^)	Continuous	99.03 ± 121.80	101.92 ± 119.06	0.808
FoxP3 + TILs (cells/mm^2^)	Continuous	159.47 ± 117.33	149.04 ± 115.16	0.363
Recurrence during follow-up	No	107	155	0.920
	Yes	66	98	
Survival during follow-up	Alive	153	218	0.557
	Dead	20	35	

Bold values indicate *P* < 0.05

*TSR* tumor-stroma ratio, *PD-1* programmed cell death protein-1, *PD-L1* programmed cell death-1, *TILs* tumor-infiltrating lymphocytes

### TSR

The distribution of TSR levels among 173 GCTB cases in the training cohort and 253 GCTB cases in the validation cohort is depicted in Supplemental Digital Content. Figure S5C. Strong correlation was observed between the two methods in terms of TSR evaluation (r = 0.934, *P* < 0.001 in the training cohort; r = 0.925, *P* < 0.001 in the validation cohort. Supplemental Digital Content. Figure S5D, F), with the Bland–Altman plot verifying that the mean difference in TSR data between the two methods was minor (*P* < 0.001, Supplemental Digital Content. Figure S5E, S5G). This result proves that simple and easy-to-operate HE-stained sections can accurately evaluate TSR.

### Influence of TSR on the survival of GCTB patients

#### Univariate Kaplan–Meier analysis: LRFS

**Fig. 1:**
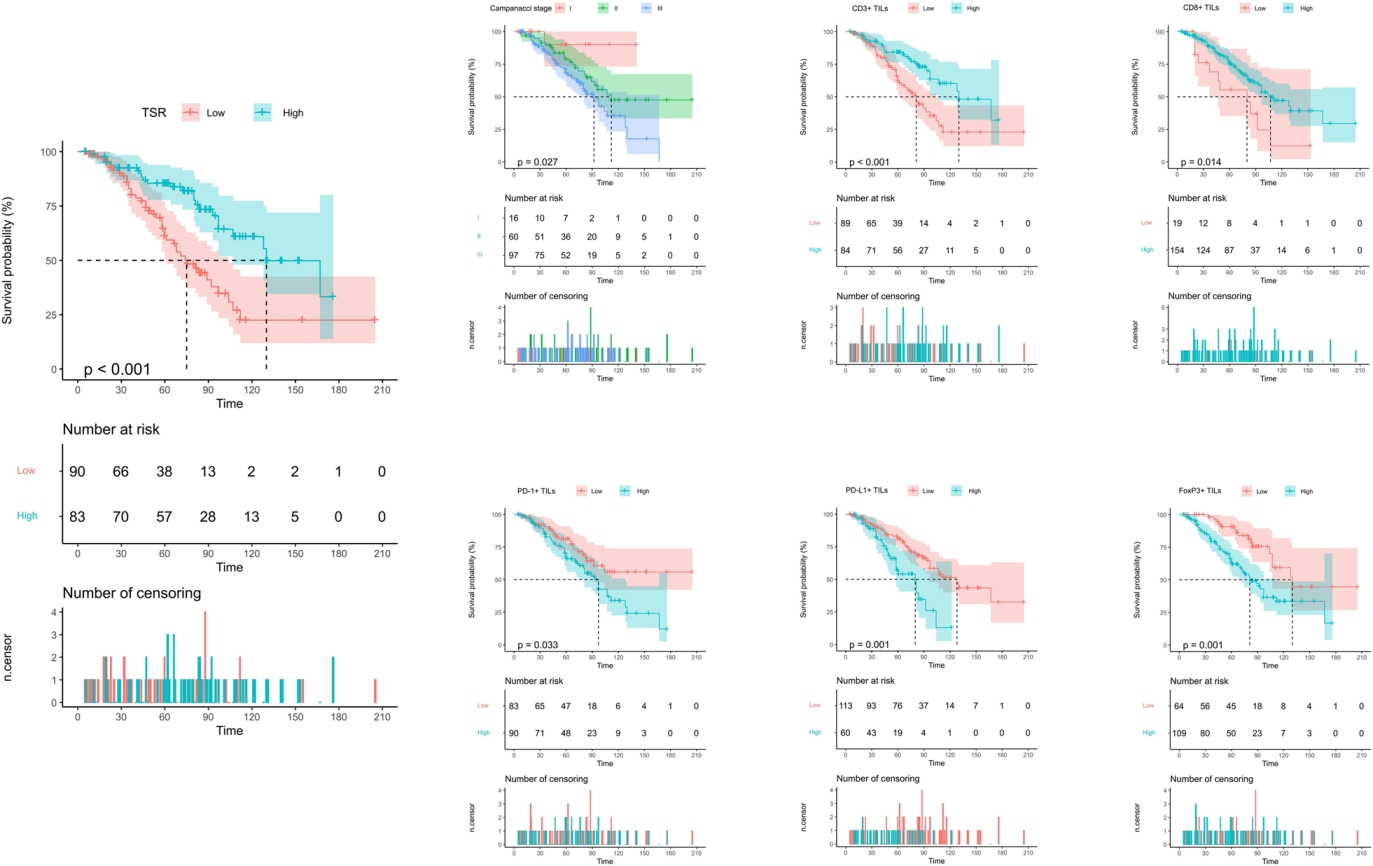
Kaplan–Meier curves of local recurrence-free survival of GCTB patients in the training cohort stratified by TSR, Campanacci staging, CD3 + TILs, CD8 + TILs, PD-1 + TILs, PD-L1 + TILs, and FoxP3 + TILs

In the training cohort, findings from the univariate Kaplan–Meier analysis revealed that low TSR was indicative of poorer LRFS in patients (*P* < 0.001, Supplemental Digital Content. Table S2, and Fig. 1). Additionally, high Campanacci staging, PD-1 + TIL, PD-L1 + TIL, and FoxP3 + TIL were all associated with inferior LRFS in patients, whereas high CD3 + TIL and CD8 + TIL were linked to better LRFS in patients (P = 0.001 and P = 0.014, respectively. Supplemental Digital Content. Table S2, and Fig. 1). Nonetheless, other variables did not demonstrate a statistically significant association with patient LRFS (Supplemental Digital Content. Table S2).

In the validation cohort, the results of univariate Kaplan–Meier analysis showed that TSR was predictive of LRFS with similar findings to the training cohort (*P* < 0.001, Supplemental Digital Content. Table S2 Supplemental Digital Content. Figure S9). Moreover, postoperative neurological dysfunction, type of resection, Campanacci stage, Ki-67 expression, CD3 + TIL, CD8 + TIL, and PD-L1 + TIL were significantly associated with patient LRFS (*P* = 0.045, *P* = 0.046, *P* = 0.007, *P* = 0.002, *P* = 0.001, *P* = 0.002 and *P* < 0.001, respectively. Supplemental Digital Content. Table S2 and Supplemental Digital Content. Figure S9). Nonetheless, other variables did not demonstrate a statistically significant association with patient LRFS (Supplemental Digital Content. Table S2).

## Univariate Kaplan–Meier analysis: OS

**Fig. 2:**
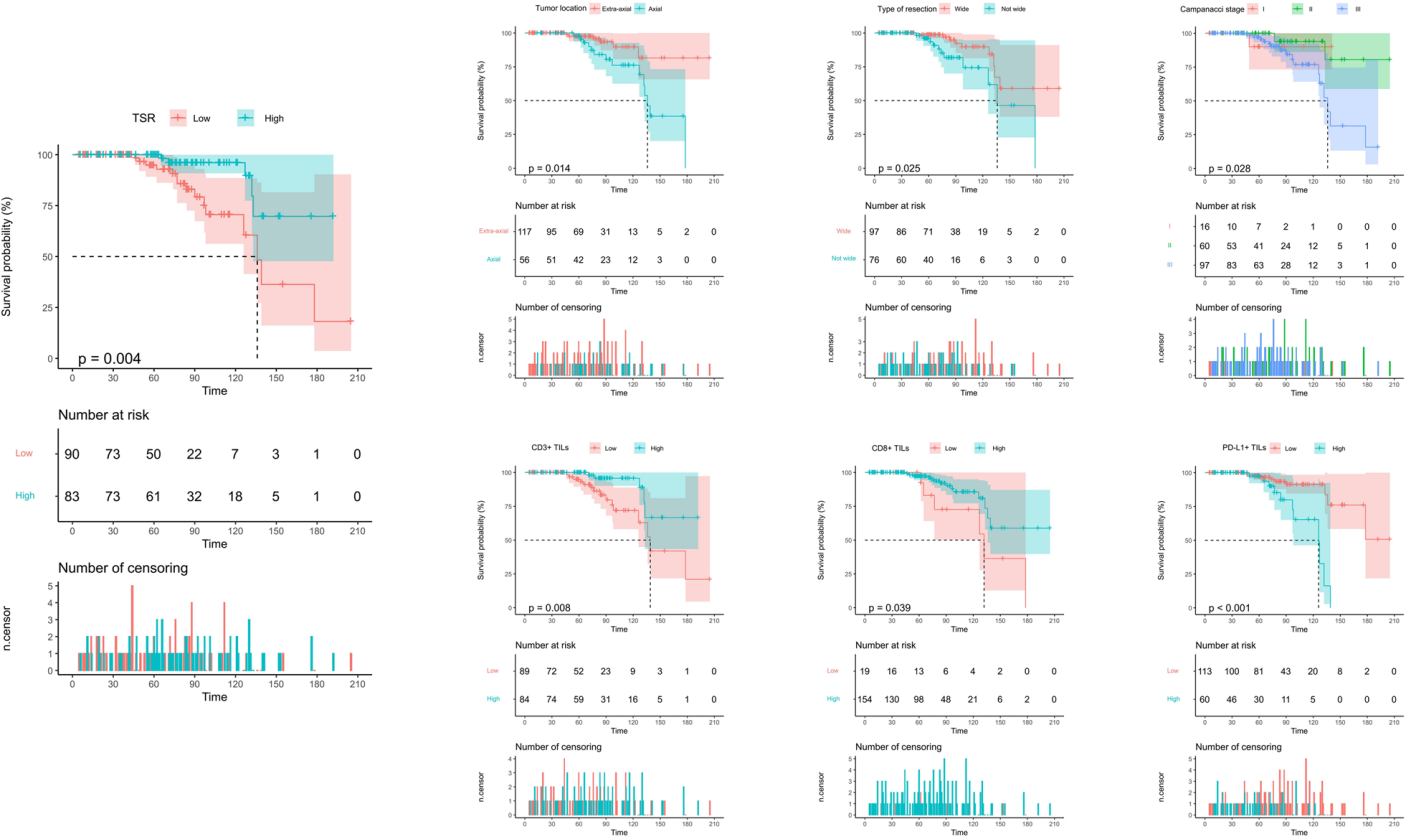
Kaplan–Meier curves of overall survival of GCTB patients in the training cohort stratified by TSR, tumor location, type of resection, Campanacci stage, CD3 + TILs, CD8 + TILs, and PD-L1 + TILs

In the training cohort, the results of univariate Kaplan–Meier analysis demonstrated that low TSR was associated with lower OS in patients (*P* = 0.004, Supplemental Digital Content. Table S3, and Fig. 2). Moreover, axial GCTB patients, rather than wide resection, high Campanacci stage, and PD-L1 + TIL were significantly associated with poor OS, while high CD3 + TIL and CD8 + TIL were associated with better OS in patients (*P* = 0.008 and P = 0.039, respectively. Supplemental Digital Content. Table S3 and Fig. 2). However, other variables did not show a statistically significant association with patient OS (Supplemental Digital Content. Table S3).

In the validation cohort, the results of univariate Kaplan–Meier analysis showed that TSR predicted OS with similar results to the training cohort (*P* < 0.001, Supplemental Digital Content. Table S3, Supplemental Digital Content. Figure S10). Additionally, tumor location, Campanacci stage, Ki-67 expression, CD3 + TIL, CD4 + TIL, CD8 + TIL, CD20 + TIL, and PD-L1 + TIL were all significantly associated with patient OS (*P* = 0.044, *P* = 0.028, *P* = 0.009, *P* < 0.001, *P* = 0.049, *P* < 0.001, *P* = 0.047 and *P* < 0.001, respectively. Supplemental Digital Content. Table S3 and Supplemental Digital Content. Figure S10). Nonetheless, other variables did not demonstrate a statistically significant association with patient OS (Supplemental Digital Content. Table S3).

### Multivariate Cox analysis

**Fig. 3:**
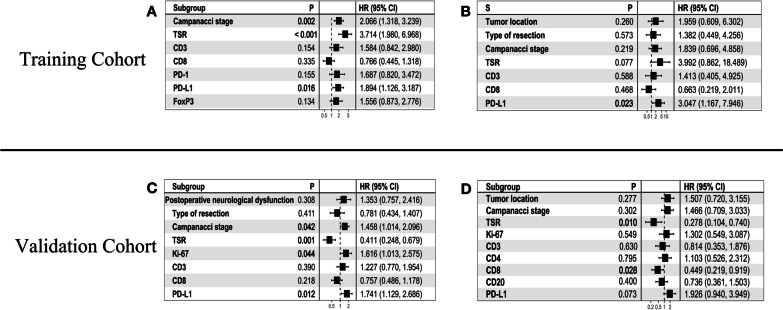
Multivariate Cox proportional hazard analyses of prognostic factors for local recurrence-free survival in patients with GCTB in the training cohort (**A**); Multivariate Cox proportional hazard analyses of prognostic factors for overall survival in patients with GCTB in the training cohort (**B**); Multivariate Cox proportional hazard analyses of prognostic factors for local recurrence-free survival in patients with GCTB in the validation cohort (**C**); Multivariate Cox proportional hazard analyses of prognostic factors for overall survival in patients with GCTB in the validation cohort (**D**)

In the training cohort, multivariate Cox analysis indicated that TSR, Campanacci stage, and PD-L1 + TILs independently predicted LRFS in patients (P < 0.001, *P* = 0.002 and *P* = 0.016, respectively. Figure 3A), while PD-L1 + TILs independently predicted patient OS (P = 0.023. Figure. 3B).

In the validation cohort, multivariate Cox analysis showed that TSR, Campanacci stage, Ki-67 + expression, and PD-L1 + TIL independently predicted LRFS in patients (*P* < 0.001, *P* = 0.042, *P* = 0.044 and *P* = 0.012, respectively. Figure 3C). TSR independently predicted OS in patients (*P* = 0.01. Figure 3D).

#### Relationship between TSR, and clinicopathological features and the immune microenvironment

**Fig. 4:**
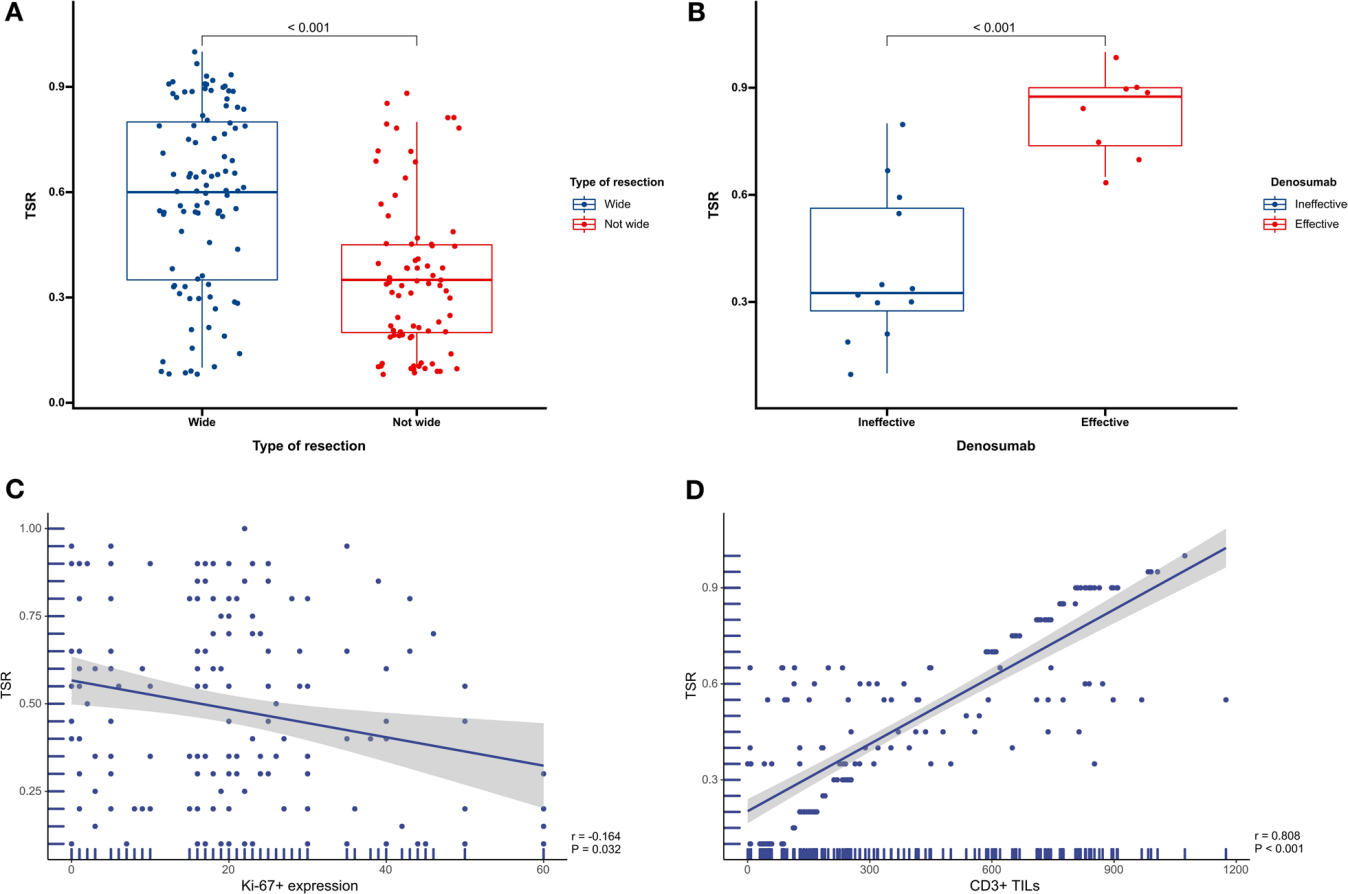
Association between TSR and type of resection in the training cohort (**A**); Association between TSR and denosumab treatment responsiveness in the training cohort (**B**); Association between TSR and Ki-67 expression in the training cohort (**C**); Association between TSR and CD3 expression in the training cohort (**D**)

In the training cohort, a significant difference was observed in TSR between the not wide resection group and the wide resection group (*P* < 0.001. Figure 4A). Moreover, TSR was positively correlated with CD3 + TIL but was found to be significantly associated with Ki-67 expression (*P* = 0.032 and *P* < 0.001, respectively. Figure 4C, D). Other variables did not exhibit a significant association with TSR (Supplemental Digital Content. Figure S11).

**Fig. 5:**
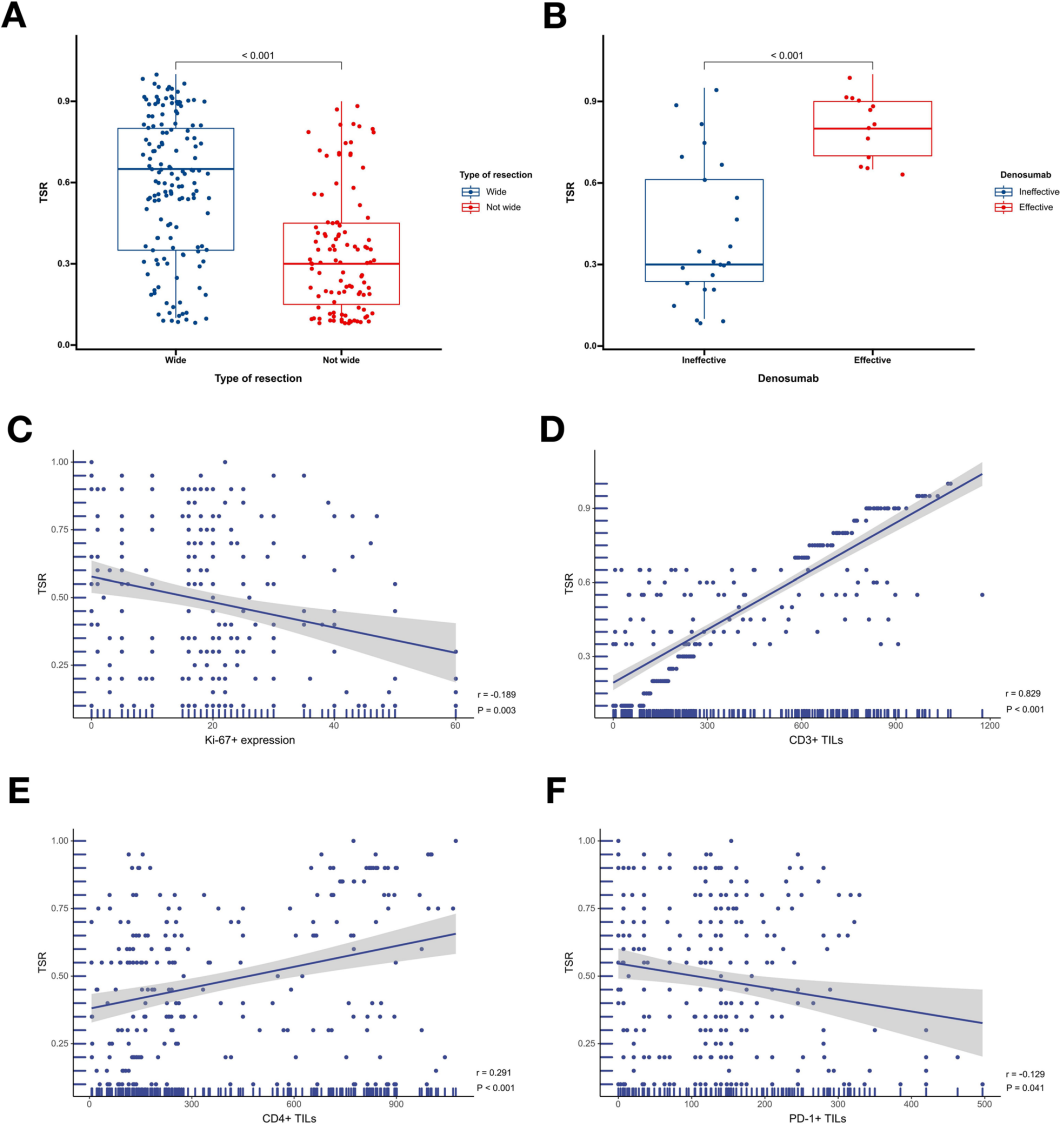
Association between TSR and type of resection in the validation cohort (**A**); Association between TSR and denosumab treatment responsiveness in the validation cohort (**B**); Association between TSR and Ki-67 expression in the validation cohort (**C**); Association between TSR and CD3 expression in the validation cohort (**D**); Association between TSR and CD4 expression in the validation cohort (**E**); Association between TSR and PD-L1 expression in the validation cohort (**F**)

Similarly, in the validation cohort, TSR was significantly higher in the wide resection group than in the not wide resection group (*P* < 0.001. Figure 5A). Additionally, TSR was positively correlated with CD3 + TIL and CD4 + TIL but negatively correlated with Ki-67 expression and PD-L1 + TIL (*P* < 0.001, *P* < 0.001, *P* = 0.003 and *P* = 0.041, respectively. Figure 5C–F). No significant associations were found with other variables (Supplemental Digital Content. Figure S12).

#### TSR, and denosumab treatment responsiveness

In the training cohort, among the 20 patients treated with denosumab, 8 showed remission of tumor symptoms and 12 showed no response. The TSR was significantly higher in the effective group than in the ineffective group (*P* < 0.001. Figure 4B). In the validation cohort, among the 37 patients treated with denosumab, 13 achieved remission of tumor symptoms and 24 did not respond to treatment. The TSR was significantly higher in the effective group compared to the ineffective group (*P* < 0.001. Figure 5B).

#### Comparison of the Campanacci staging system and TSR in outcome prediction

**Fig. 6:**
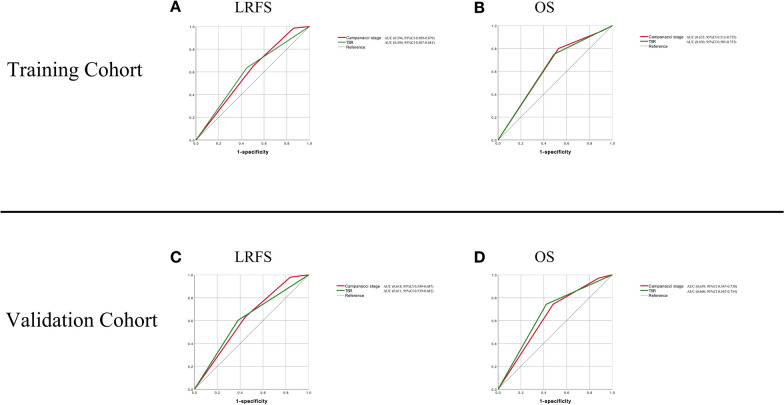
Comparison of the sensitivity and specificity between TSR and the Campanacci staging system for local recurrence-free survival prediction in the training cohort (**A**); Comparison of the sensitivity and specificity between TSR and the Campanacci staging system for overall survival prediction in the training cohort (**B**); Comparison of the sensitivity and specificity between TSR and the Campanacci staging system for local recurrence-free survival prediction in the validation cohort (**C**); Comparison of the sensitivity and specificity between TSR and the Campanacci staging system for overall survival prediction in the validation cohort (**D**)

In both cohorts, TSR was a comparable predictor of LRFS and OS along with the Campanacci stage (Figure 6A–D).

## Discussion

### Key results

In recent years, scholars have conducted extensive research to assess the potential impact of the tumor microenvironment on tumor progression. Among these investigations, TSR has garnered increasing attention. To date, although the precise role of TSR in tumor prognosis remains controversial [8, 9], a substantial body of evidence suggests that TSR serves as an independent predictor of adverse outcomes [9], a finding consistent with our research results. In this investigation, we characterized the TSR in GCTB and examined its associations with various clinical factors, immune microenvironment, and response to denosumab therapy. We discovered that both TSR measures were correlated with patient outcomes in terms of LRFS and OS in both patient cohorts, and TSR serves as an independent predictor of poor prognosis for both LRFS and OS. Furthermore, they were also significantly associated with multiple clinicopathological features, expression of TILs, and denosumab treatment responsiveness. Notably, both TSR measures were found to be comparable with the traditional Campanacci staging system in their ability to predict patient outcomes. These findings indicate that TSR plays a crucial role in the progression of GCTB, patient immune response, and response to treatment. In addition, the simple and easy-to-operate HE-stained sections can accurately evaluate TSR without resorting to other complicated operations. In the future, the combination of targeted drugs for the tumor stroma and immune modulators may offer novel therapeutic options and facilitate the prediction of clinical response to denosumab therapy.

### Relationship between the TSR and prognosis, clinicopathological features, and immune microenvironment

The assessment of the immune microenvironment's potential impact on tumor progression has brought increasing attention to the TSR. While the precise role of the TSR in tumor prognosis remains a subject of controversy [8, 26], abundant evidence suggests that a low TSR is an independent factor for poor prognosis [9, 26], which is consistent with our findings. Stromal components play a crucial role in facilitating communication between stromal and cancer cells, and fibroblasts, among other stromal cells, can accelerate tumor development and metastasis [8]. Moreover, our study demonstrated that patients in the wide resection group had a significantly higher TSR than those in the not wide resection group. This finding suggests that tumors with a low TSR are generally more aggressive [27–29], thus making surgery more challenging.

Additionally, the TSR of patients in the CD3 + TILs high expression group was significantly higher than that of the CD3 + TILs low expression group. The high expression of CD3 + TILs and CD8 + TILs have been shown to predict a favorable prognosis in GCTB patients, which is consistent with previous reports [30, 31]. This result implies that the tumor stroma or its components may affect GCTB progression by inhibiting the infiltration of immune effector cells and promoting the accumulation of regulatory T cells at the tumor site. Current clinical research and trials demonstrate that drugs targeting the tumor stroma have achieved clinical efficacy in treating various tumors [32–34], thus providing new insights for developing new treatment options for GCTB.

Previously, it has been reported that the PD-1/PD-L1 signaling pathway can induce tumor immune evasion by inhibiting T cell function, thereby contributing to poor prognosis in GCTB [6]. This finding is consistent with our research results, which demonstrate that high expression of PD-1 and PD-L1 worsens progression-free survival and OS in GCTB patients. Additionally, there has been ongoing debate regarding the role of FoxP3 in tumor development, whether it acts as a promoter or inhibitor [35, 36]. Our study found that high expression of FoxP3 correlates with poorer PFS in patients. It has been reported that FoxP3, similar to the function of the PD-1/PD-L1 signaling pathway, may also inhibit T cell function and participate in immune evasion, thereby promoting cancer initiation and progression, while also contributing to resistance to targeted therapies such as PD-1/PD-L1 inhibitors [37, 38].

Furthermore, high Ki-67+ expression is a prognostic or predictive marker for various malignancies, indicating higher-grade lesions and aggressiveness [39]. Our study found that the TSR was negatively correlated with Ki-67+ expression, further corroborating that a low TSR may exhibit similar biological behavior to high Ki-67+ expression, ultimately resulting in a worse prognosis for GCTB patients.

### Relationship between TSR and the therapeutic responsiveness of denosumab

The present study revealed that a low TSR could lead to denosumab treatment resistance in GCTB patients. However, treatment resistance is not solely attributed to cancer cells, as the tumor-associated stroma also contributes to this phenomenon by promoting tumor progression through various molecular mechanisms [14]. Denosumab functions as a RANKL inhibitor, which suppresses bone resorption by blocking RANK-RANKL binding, and eliminates multinucleated osteoclast-like giant cells to achieve therapeutic efficacy against GCTB [4]. Nevertheless, stromal cells within the interstitium can overexpress RANKL, which not only facilitates monocyte precursor recruitment but also significantly increases the number of multinucleated osteoclast-like giant cells [40]. This could explain why patients with low TSR respond poorly to denosumab therapy.

### Limitations

To minimize the heterogeneity of each variable group, we simplified the grouping criteria in the statistical analysis to enhance the reliability of the results. Nonetheless, this retrospective study still has limitations, and future prospective randomized clinical trials are necessary to verify our current conclusions. As an exploratory correlation study, further investigations are warranted to unravel the specific molecular mechanisms of TSR that influence patient clinical outcomes, immune microenvironment, and response to denosumab treatment. Furthermore, the number of patients included in our study who received denosumab postoperatively was limited. However, we believe that future well-designed prospective large cohorts' clinical studies will help elucidate the relationship between TSR and the response to denosumab treatment in unresectable or advanced GCTB cases.

## Conclusion

This is the first multicenter large cohort study on TSR in GCTB, and it is evident that TSR correlates with the clinicopathological features, immune microenvironmental parameters, and denosumab treatment responsiveness in GCTB patients. A low TSR is an independent predictor of a worse prognosis in terms of LRFS and OS and may also indicate denosumab treatment resistance. Furthermore, TSR is comparable to the traditional Campanacci staging system in predicting prognosis, but international and standardized TSR scoring systems need further development to enhance their accuracy.

### Supplementary Information

Supplementary file 1.

## Data Availability

The datasets generated during and/or analysed during the current study are available from the corresponding author on reasonable request.
