# Supplementary material for: The tumor-stroma ratio in giant cell tumor of bone: associations with the immune microenvironment and responsiveness to denosumab treatment
Source: J Orthop Surg Res. 2024 Jul 15;19:405. doi: 10.1186/s13018-024-04885-8 (PMC11250954; doi:10.1186/s13018-024-04885-8)
Supplement: Supplementary file 1 [file 13018_2024_4885_MOESM1_ESM.pdf]

Supplemental Digital Content. Table 1. Detailed information on the primary antibodies used for the immunohistochemistry and Immunofluorescence assay.

Antibody	Host species	Description	Clone number	Catalog No.	Company	Dilution
Ki-67	Mouse	monoclonal	MAB107	ZM-0166	ZSGB-BIO	1:100
CD3	Mouse	monoclonal	LN10	ZM-0417	ZSGB-BIO	1:100
CD4	Mouse	monoclonal	UMAB64	ZM-0418	ZSGB-BIO	1:100
CD8	Rabbit	monoclonal	SP16	ZA-0508	ZSGB-BIO	1:100
CD20	Rabbit	monoclonal	EP459Y	Ab78237	Abcam	1:100
PD-1	Mouse	monoclonal	UMAB199	ZM-0381	ZSGB-BIO	1:50
PD-L1	Rabbit	monoclonal	E1L3N	CST13684	Cell Signaling Technology	1:100
FoxP3	Mouse	monoclonal	236A/E7	Ab20034	Abcam	1:100
H3.3G34W	Rabbit	monoclonal	EPR23581-39	Ab272691	Abcam	1:100

PD-1, programmed cell death protein 1; PD-L1, programmed cell death-1;

Supplemental Digital Content. Table 2. Univariate analysis of the prognostic factors of local recurrence-free survival in patients with GCTB

Factors	Categories	Training Cohort (n = 173)			Validation Cohort (n = 253)		
		Number of patients	Univariate analysis		Number of patients	Univariate analysis	
			χ^2	*P*-value		χ^2	*P*-value
Age (years)	Young (≤ cutoff[a])	96	1.175	0.278[b]	85	0.787	0.375[b]
	Old (> cutoff[a])	77			168		
Gender	Female	61	0.282	0.596	99	0.767	0.381
	Male	112			154		
Tumor location	Extra-axial	117	3.759	0.053	183	2.058	0.151
	Axial	56			70		
Duration of symptoms (months)	Short (≤ cutoff[a])	69	0.027	0.870[b]	176	0.000	0.997[b]
	Long (> cutoff[a])	104			77		
Preoperative neurological dysfunction	No	136	0.042	0.837	208	0.311	0.577
	Yes	37			45		
Postoperative neurological dysfunction	No	119	3.661	0.056	186	4.019	**0.045**
	Yes	54			67		
Tumor size (in diameter, cm)	Small (≤ cutoff[a])	86	0.270	0.603[b]	20	0.086	0.769[b]
	Large (> cutoff[a])	87			223		
Type of resection	Wide	97	3.124	0.077	147	3.975	**0.046**
	Not wide	76			106		

Campanacci stage	I	16	7.211	**0.027**	27	9.826	**0.007**
	II	60			95		
	III	97			131		
TSR	Low (≤ cutoff[a])	90	13.786	**< 0.001**[b]	118	18.778	**< 0.001**[b]
	High (> cutoff[a])	83			135		
Ki-67 expression	Low (≤ cutoff[a])	124	1.488	0.223[b]	69	9.223	**0.002**[b]
	High (> cutoff[a])	49			184		
CD3 +TILs	Low (≤ cutoff[a])	89	11.324	**0.001**[b]	109	10.762	**0.001**[b]
	High (> cutoff[a])	84			144		
CD4 +TILs	Low (≤ cutoff[a])	43	2.661	0.103[b]	28	0.801	0.371[b]
	High (> cutoff[a])	130			225		
CD8 +TILs	Low (≤ cutoff[a])	19	6.067	**0.014**[b]	109	9.558	**0.002**[b]
	High (> cutoff[a])	154			144		
CD20 +TILs	Low (≤ cutoff[a])	77	0.009	0.926[b]	113	0.022	0.883[b]
	High (> cutoff[a])	96			140		
PD-1 +TILs	Low (≤ cutoff[a])	83	4.569	**0.033**[b]	160	3.238	0.072[b]
	High (> cutoff[a])	90			93		
PD-L1 +TILs	Low (≤ cutoff[a])	113	11.844	**0.001**[b]	166	13.253	**< 0.001**[b]
	High (> cutoff[a])	60			87		
FoxP3 +TILs	Low (≤ cutoff[a])	64	11.204	**0.001**[b]	219	2.228	0.130[b]
	High (> cutoff[a])	109			34		

Bold values indicate $P < 0.05$; TSR, tumor-stroma ratio; PD-1, programmed cell death protein-1; PD-L1, programmed cell death-1; [a]Cutoff points were obtained by the X-tile software; Cutoff points for patient in training cohort TSR, age, duration of symptoms, tumor size, Ki-67 expression, CD3 +TILs, CD4 +TILs, CD8 +TILs, CD20 +TILs, PD-1 +TILs, PD-L1 +TILs, and FoxP3 +TILs in the survival analysis of OS were 0.5, 36, 8, 4.0, 24, 310, 125, 3.0, 117, 126, 120, 125 respectively. Cutoff points for patient in validation cohort TSR, age, duration of symptoms, tumor size, Ki-67 expression, CD3 +TILs, CD4 +TILs, CD8 +TILs, CD20 +TILs, PD-1 +TILs, PD-L1 +TILs, and FoxP3 +TILs in the survival analysis of OS were 0.45, 23, 11, 2.0, 10, 232, 84, 179, 118, 150, 120, 281 respectively. [b]*P* value from the log-rank test was corrected as previously suggested.

Supplemental Digital Content. Table 3. Univariate analysis of the prognostic factors of overall survival in patients with GCTB

Factors	Categories	Training Cohort (n = 173)			Validation Cohort (n = 253)		
		Number of patients	Univariate analysis		Number of patients	Univariate analysis	
			χ^2	*P*-value		χ^2	*P*-value
Age (years)	Young (≤ cutoff[a])	96	0.719	0.397[b]	85	2.434	0.119[b]
	Old (> cutoff[a])	77			168		
Gender	Female	61	0.143	0.705	99	2.064	0.151
	Male	112			154		
Tumor location	Extra-axial	117	6.089	**0.014**	183	4.016	**0.044**
	Axial	56			70		
Duration of symptoms (months)	Short (≤ cutoff[a])	69	1.683	0.194[b]	176	1.447	0.229[b]
	Long (> cutoff[a])	104			77		
Preoperative neurological dysfunction	No	136	0.356	0.551	208	0.619	0.431
	Yes	37			45		
Postoperative neurological dysfunction	No	119	3.769	0.052	186	1.818	0.178
	Yes	54			67		
Tumor size (in diameter, cm)	Small (≤ cutoff[a])	86	0.736	0.391[b]	30	3.054	0.081[b]
	Large (> cutoff[a])	87			223		
Type of resection	Wide	97	5.020	**0.025**	147	3.583	0.058
	Not wide	76			106		

Campanacci stage	I	16	7.136	**0.028**	27	7.161	**0.028**
	II	60			95		
	III	97			131		
TSR	Low (≤ cutoff[a])	90	8.384	**0.004**[b]	118	16.543	**< 0.001**[b]
	High (> cutoff[a])	83			135		
Ki-67 expression	Low (≤ cutoff[a])	124	1.849	0.174[b]	69	6.911	**0.009**[b]
	High (> cutoff[a])	49			184		
CD3 +TILs	Low (≤ cutoff[a])	89	7.059	**0.008**[b]	109	14.985	**< 0.001**[b]
	High (> cutoff[a])	84			144		
CD4 +TILs	Low (≤ cutoff[a])	43	2.202	0.138[b]	28	3.862	**0.049**[b]
	High (> cutoff[a])	130			225		
CD8 +TILs	Low (≤ cutoff[a])	19	4.267	**0.039**[b]	109	12.286	**< 0.001**[b]
	High (> cutoff[a])	154			144		
CD20 +TILs	Low (≤ cutoff[a])	77	2.994	0.084[b]	113	3.928	**0.047**[b]
	High (> cutoff[a])	96			140		
PD-1 +TILs	Low (≤ cutoff[a])	83	0.346	0.556[b]	160	3.319	0.068[b]
	High (> cutoff[a])	90			93		
PD-L1 +TILs	Low (≤ cutoff[a])	113	15.183	**< 0.001**[b]	166	16.234	**< 0.001**[b]
	High (> cutoff[a])	60			87		
FoxP3 +TILs	Low (≤ cutoff[a])	64	0.945	0.331[b]	219	3.243	0.072[b]
	High (> cutoff[a])	109			34		

Bold values indicate $P < 0.05$; TSR, tumor-stroma ratio; PD-1, programmed cell death protein-1; PD-L1, programmed cell death-1; [a]Cutoff points were obtained by the X-tile software; Cutoff points for patient in training cohort TSR, age, duration of symptoms, tumor size, Ki-67 expression, CD3 +TILs, CD4 +TILs, CD8 +TILs, CD20 +TILs, PD-1 +TILs, PD-L1 +TILs, and FoxP3 +TILs in the survival analysis of OS were 0.5, 36, 8, 4.0, 24, 310, 125, 3.0, 117, 126, 120, 125 respectively. Cutoff points for patient in validation cohort TSR, age, duration of symptoms, tumor size, Ki-67 expression, CD3 +TILs, CD4 +TILs, CD8 +TILs, CD20 +TILs, PD-1 +TILs, PD-L1 +TILs, and FoxP3 +TILs in the survival analysis of OS were 0.45, 23, 11, 2.0, 10, 232, 84, 179, 118, 150, 120, 281 respectively. [b]*P* value from the log-rank test was corrected as previously suggested.

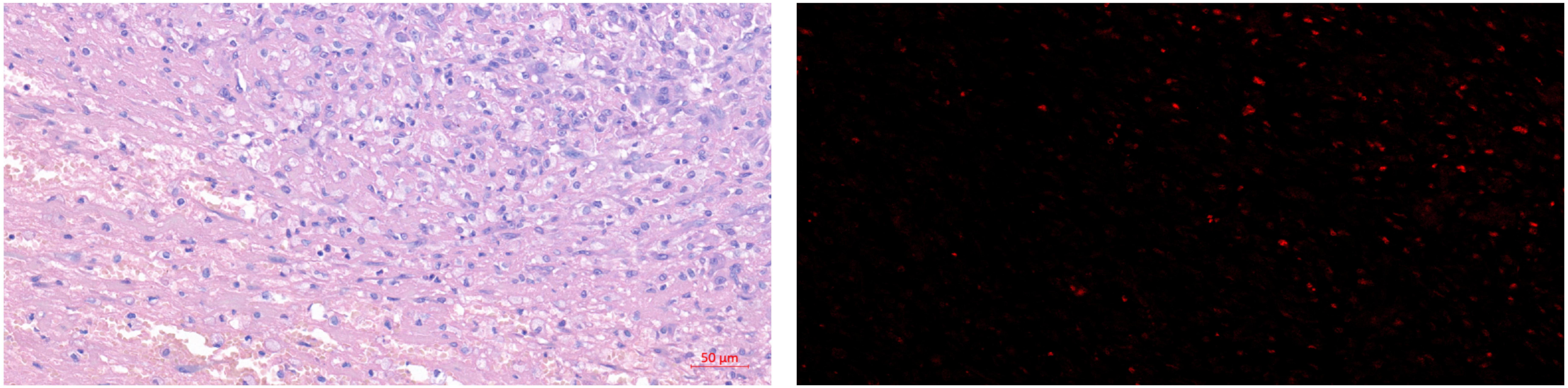

HE IF

Supplemental Digital Content. Figure 1. Representative images of hematoxylin and eosin-stained (HE) and Immunofluorescence (IF) of H3.3G34W.

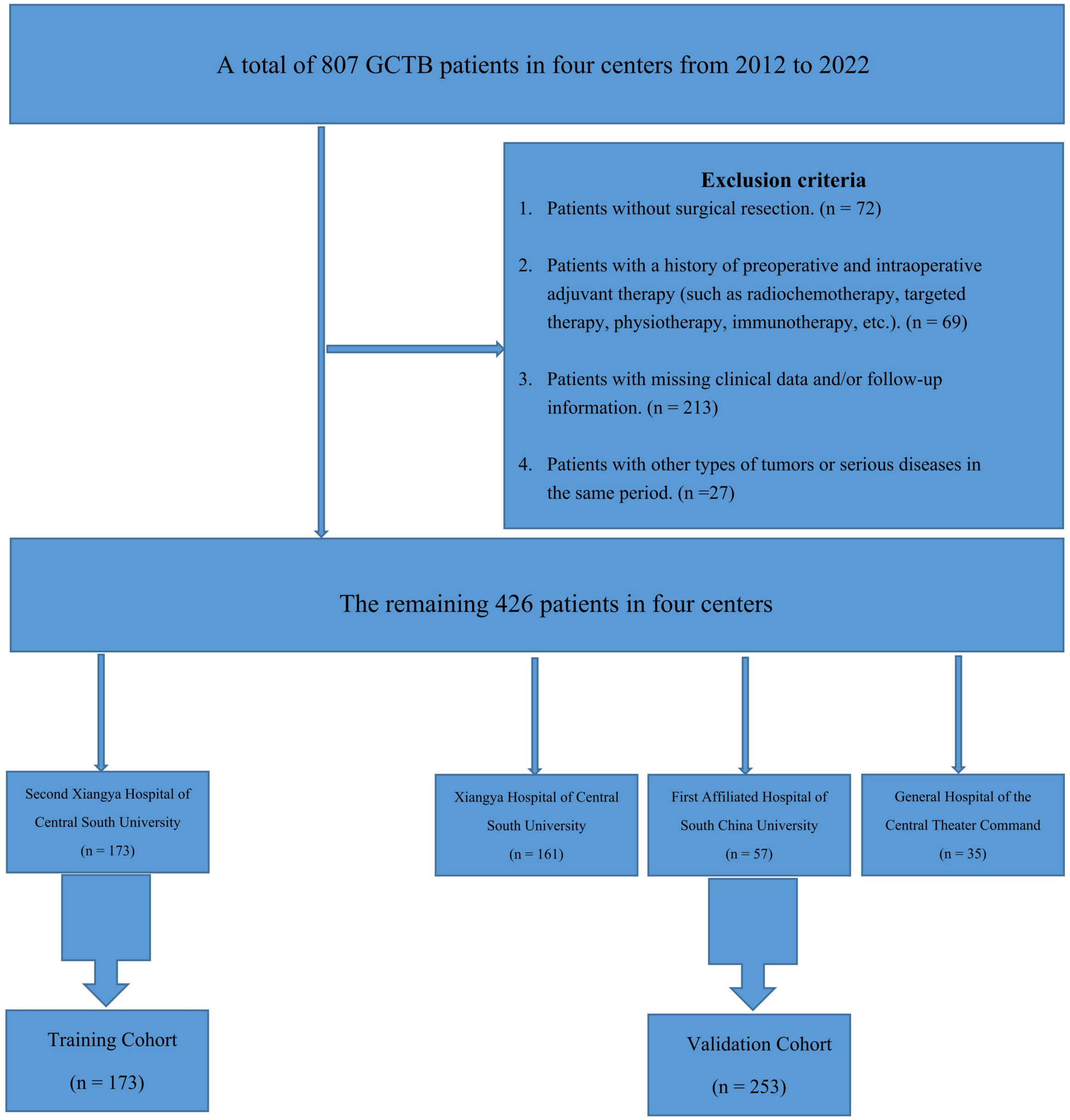

Supplemental Digital Content. Figure 2. Flow diagram of the multicenter study showing exclusion criteria and cohort settings.

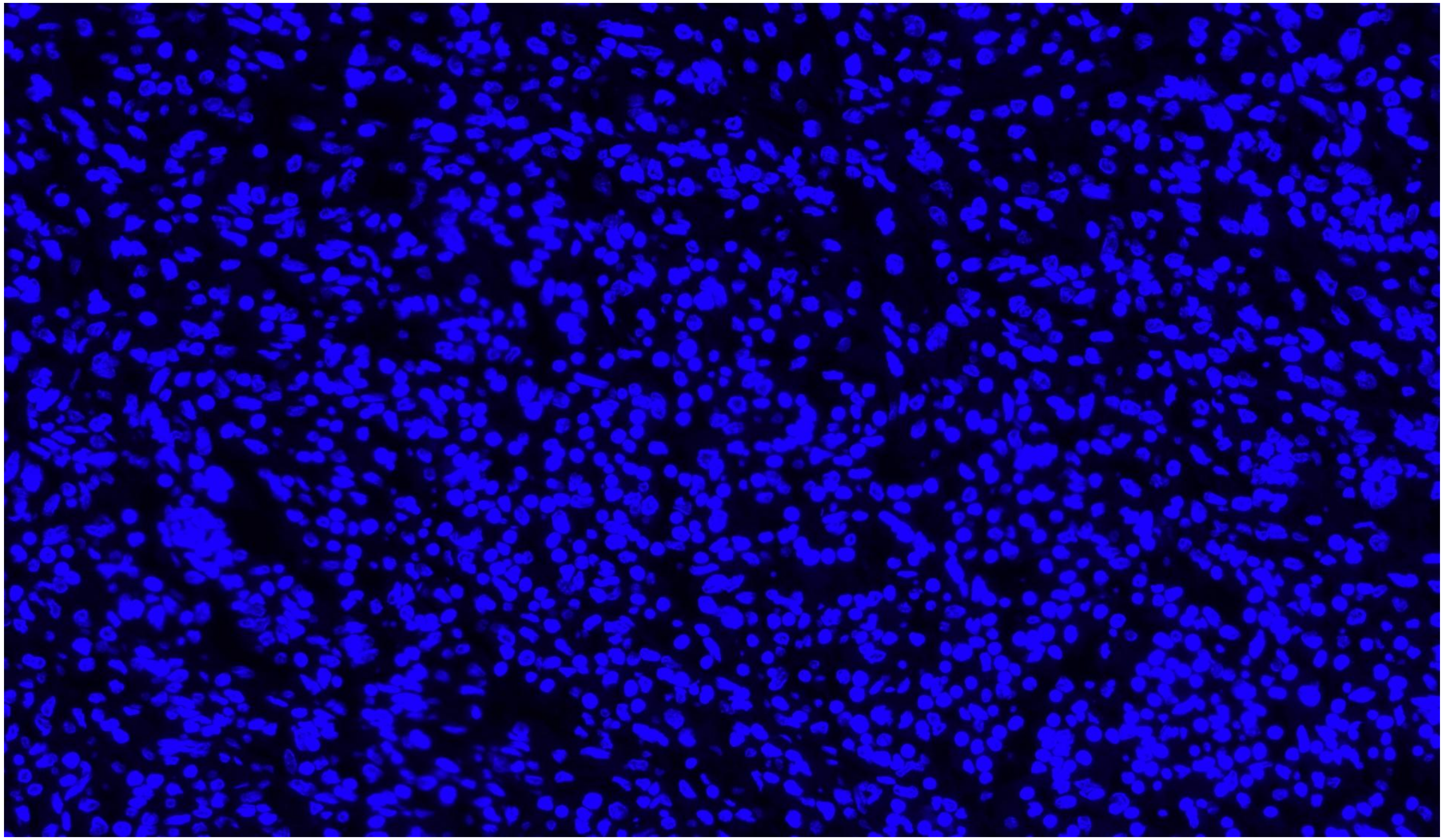

Supplemental Digital Content. Figure 3. Representative images of 4′,6-diamidino-2-phenylindole (DAPI)

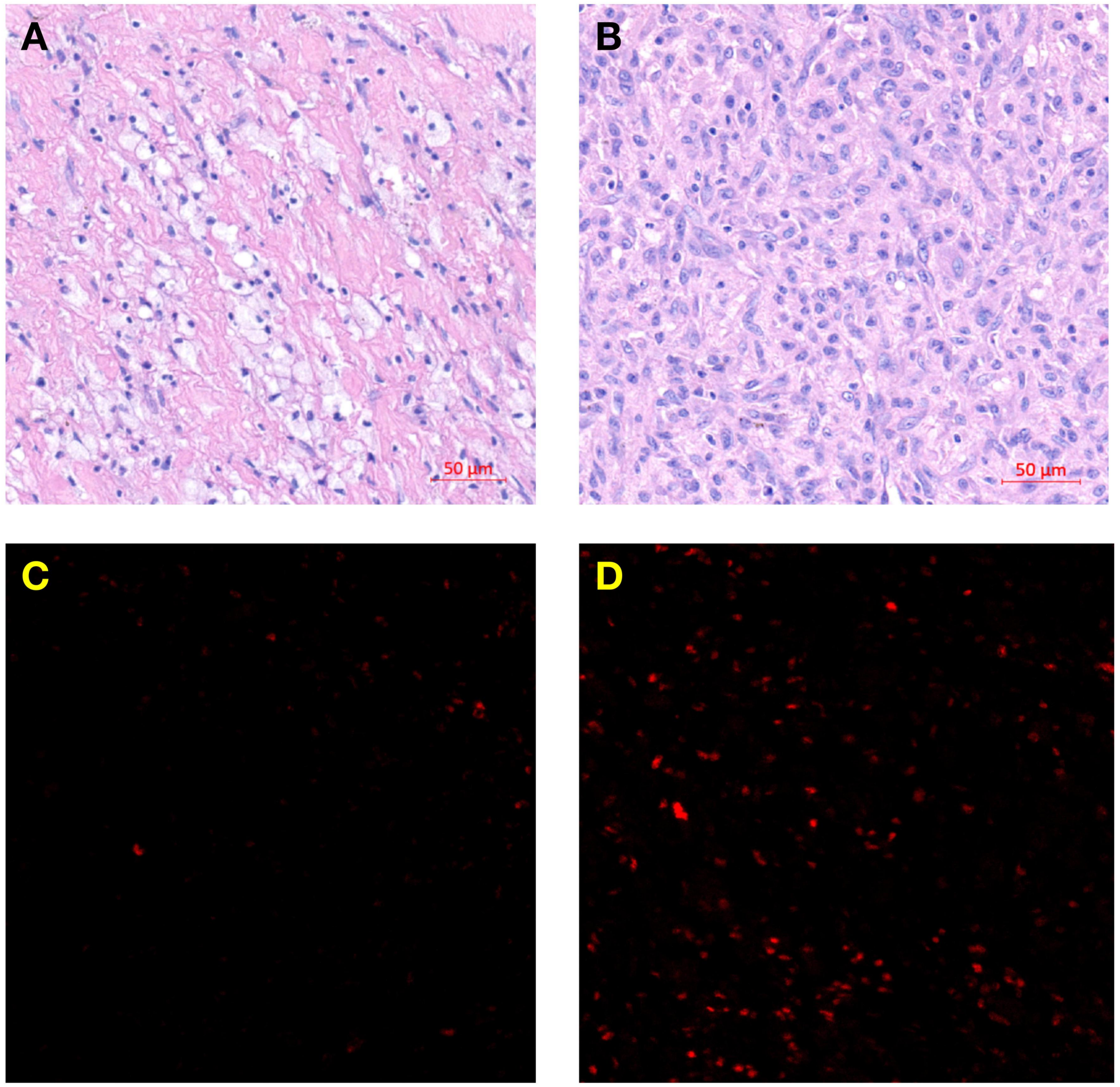

Supplemental Digital Content. Figure 4. Representative HE staining images of low tumor-stromal ratio (A); Representative HE staining images of high tumor-stromal ratio (B); Representative IF staining images of low tumor-stromal ratio (C); Representative IF staining images of high tumor-stromal ratio (D).

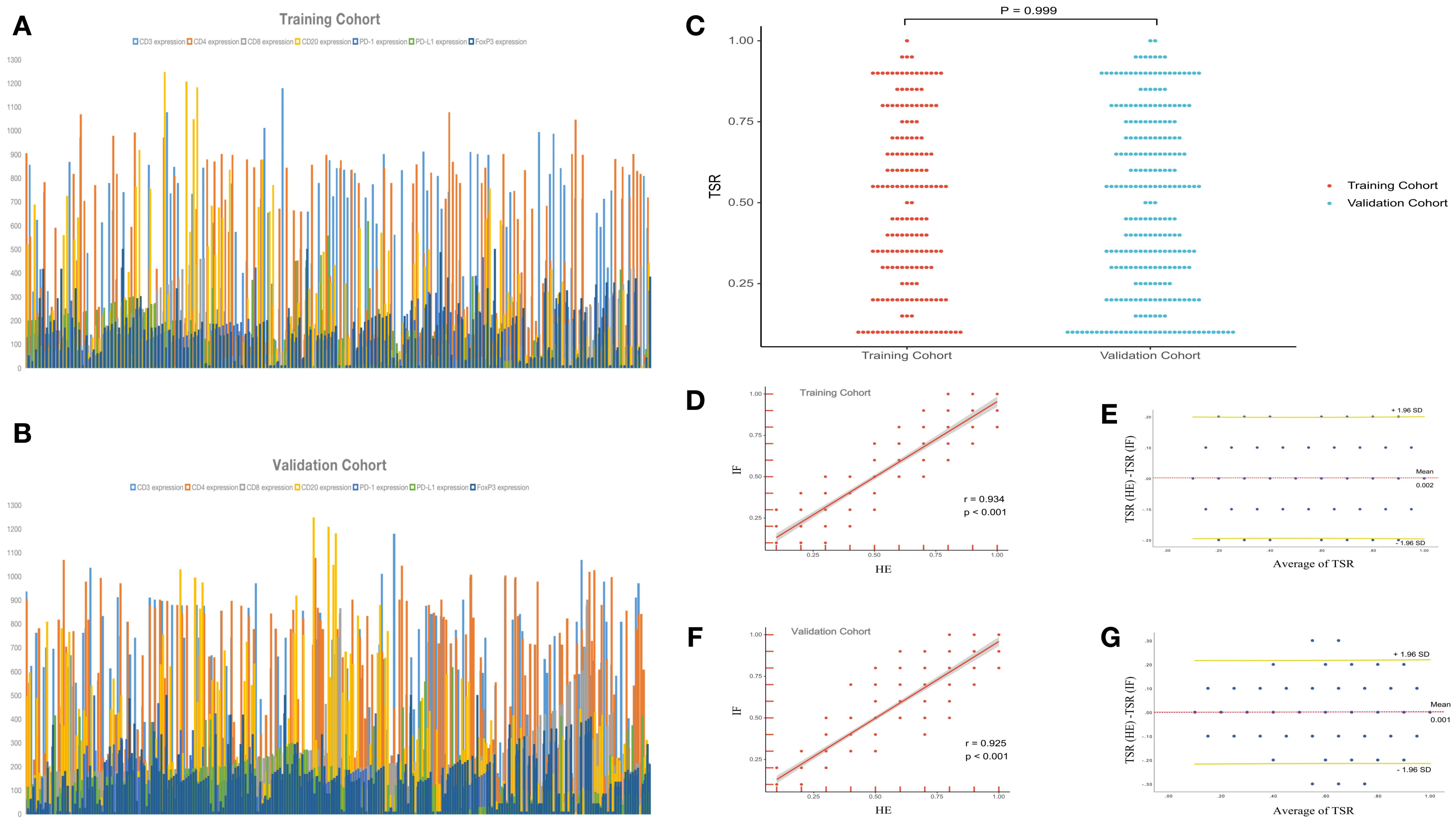

Supplemental Digital Content. Figure 5. Density of tumor-infiltrating lymphocytes (specifically including CD3+, CD4+, CD8+, CD20+, PD-1+, PD-L1+, and FoxP3+) in the training cohort (A); Density of tumor-infiltrating lymphocytes (specifically including of CD3+, CD4+, CD8+, CD20+, PD-1+, PD-L1+, and FoxP3+) in the validation cohort (B); Distribution of TSR in the two cohorts (C); Association of TSR data from 2 methods via Pearson correlation test in the training cohort (D); Bland Altman plot for agreement of TSR data from two methods in the training cohort (E); Association of TSR data from 2 methods via Pearson correlation test in the validation cohort (F); Bland Altman plot for agreement of TSR data from two methods in the validation cohort (G)

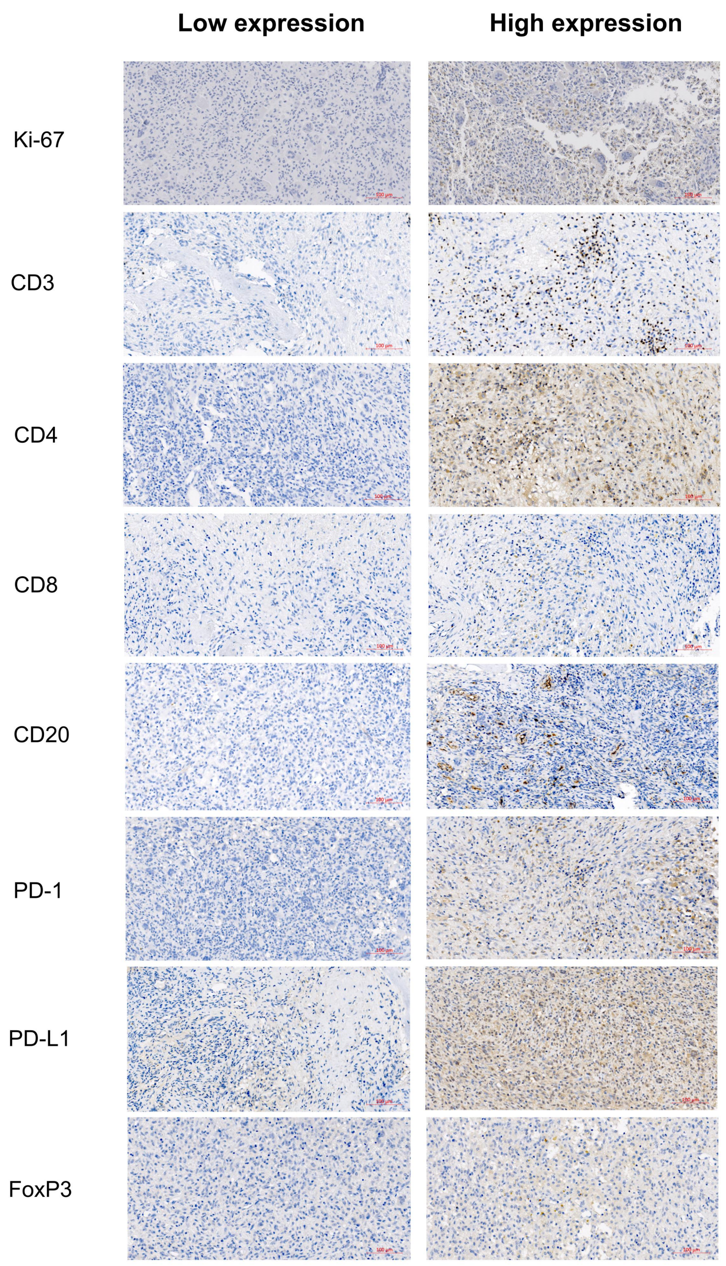

Supplemental Digital Content. Figure 6. Representative images of Ki-67 expression, CD3 +TILs, CD4 +TILs, CD8 +TILs, CD20 +TILs, PD-1 +TILs, PD-L1 +TILs, and FoxP3 +TILs in GCTB tissues.

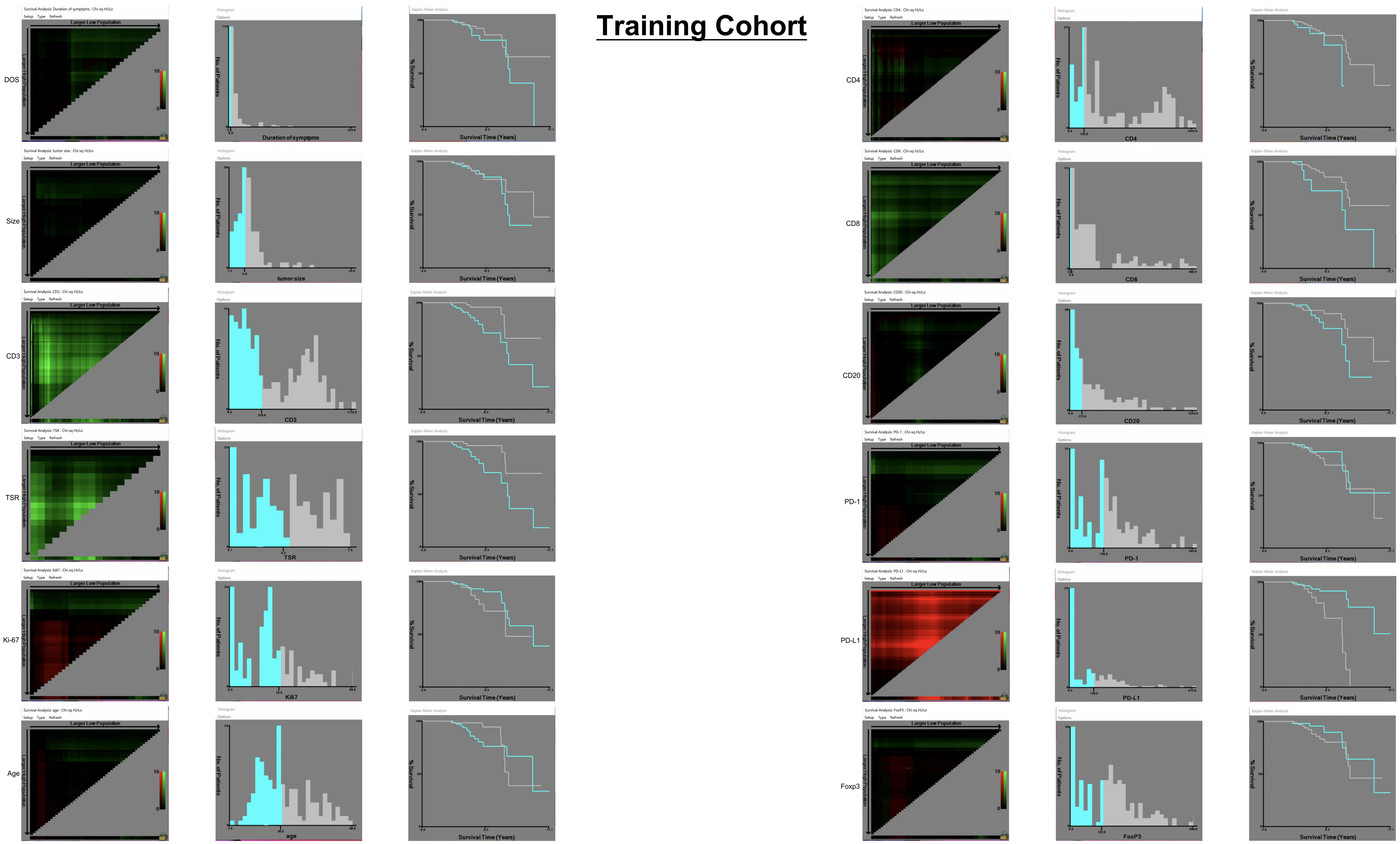

Supplemental Digital Content. Figure 7. Detailed determination process of the cutoff values for TSR, age, duration of symptoms, tumor size, Ki-67 expression, CD3 +TILs, CD4 +TILs, CD8 +TILs, CD20 +TILs, PD-1 +TILs, PD-L1 +TILs, and FoxP3 +TILs in prognosis analysis of OS in the training cohort. The cutoff value is obtained by using X-tile software, defining as the point with the minimum log-rank *P* value that was subsequently corrected according to the previously described method.

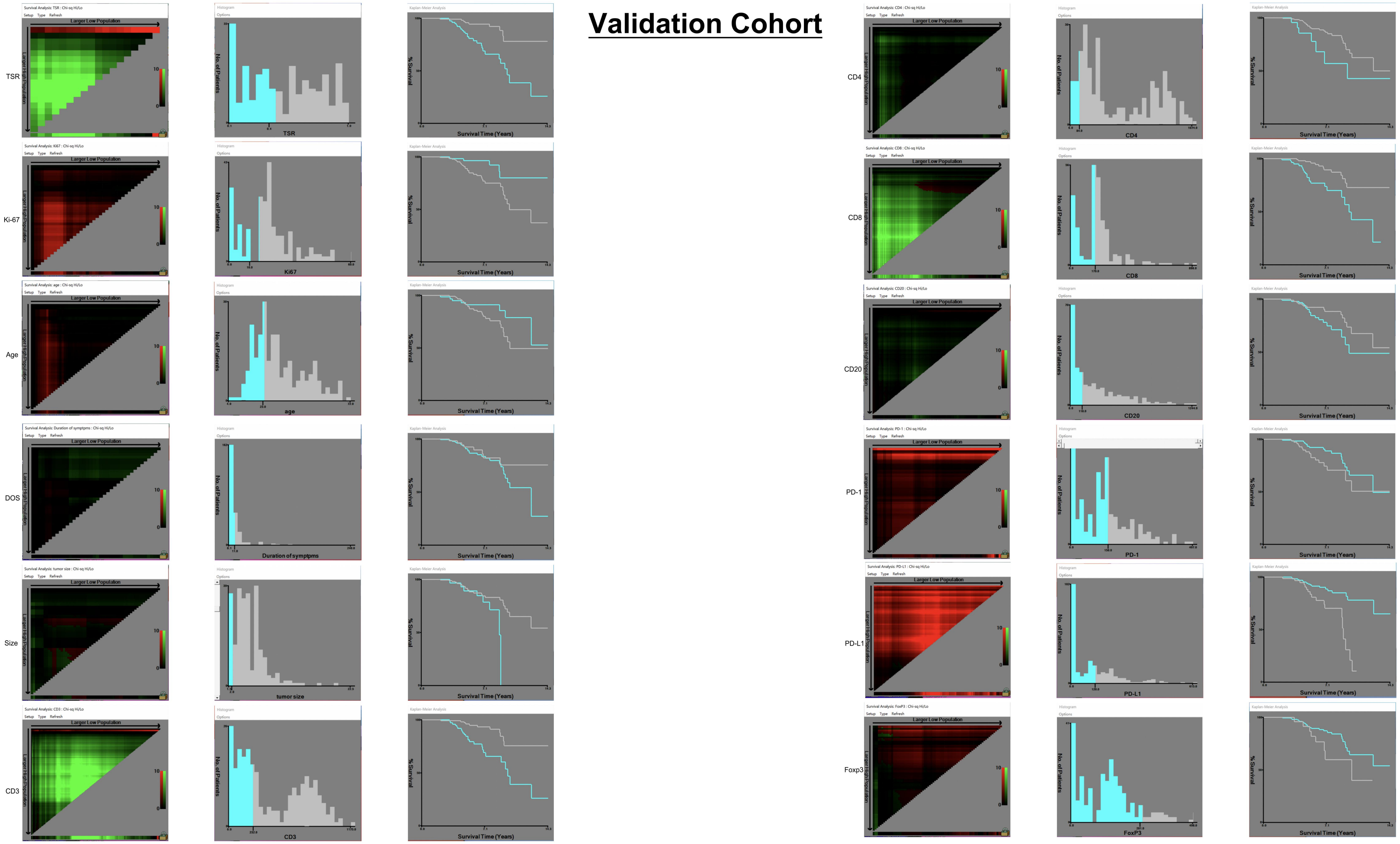

Supplemental Digital Content. Figure 8. Detailed determination process of the cutoff values for TSR, age, duration of symptoms, tumor size, Ki-67 expression, CD3 +TILs, CD4 +TILs, CD8 +TILs, CD20 +TILs, PD-1 +TILs, PD-L1 +TILs, and FoxP3 +TILs in prognosis analysis of OS in the validation cohort. The cutoff value is obtained by using X-tile software, defining as the point with the minimum log-rank *P* value that was subsequently corrected according to the previously described method.

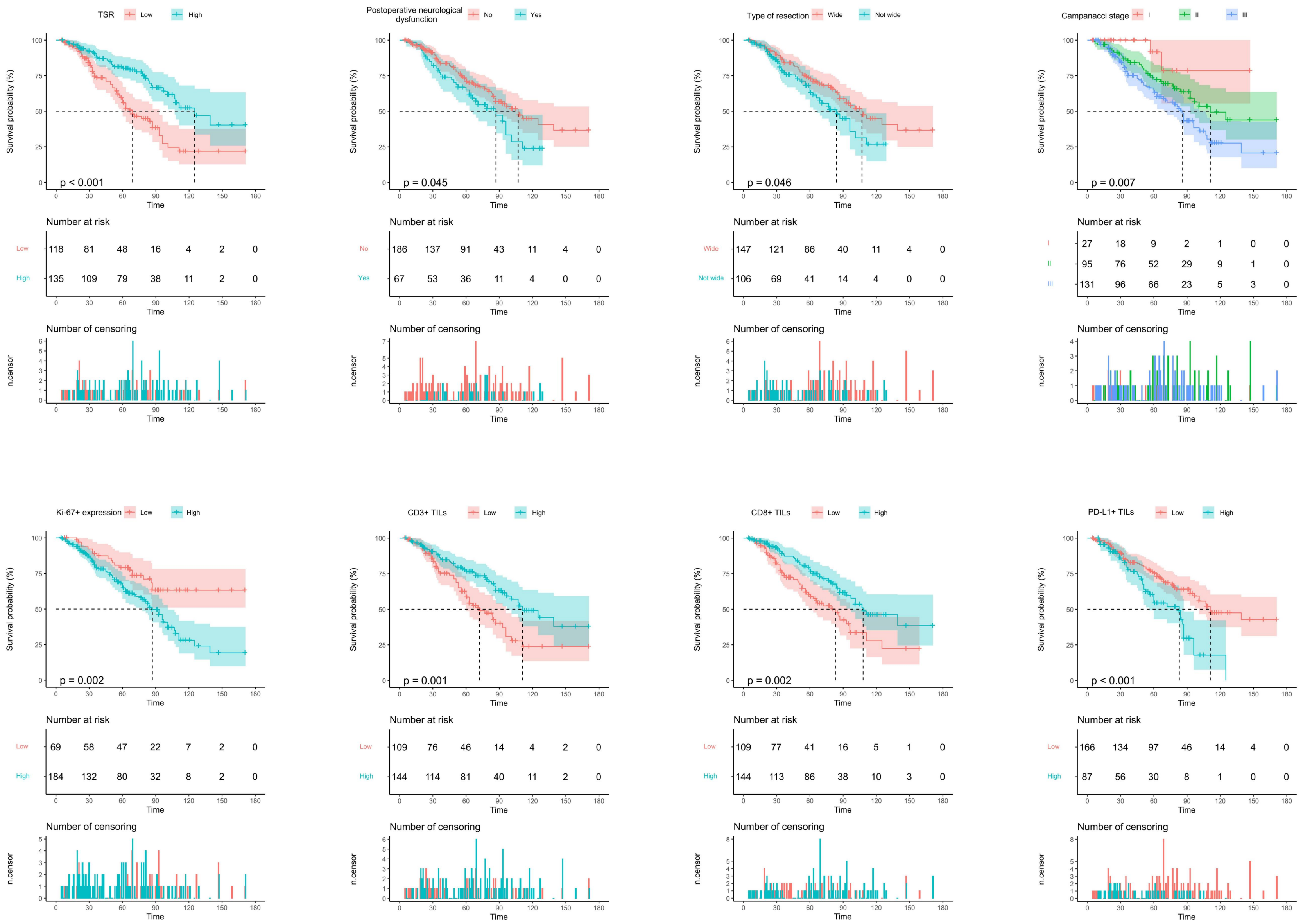

Supplemental Digital Content. Figure 9. Kaplan-Meier curves of local recurrence-free survival of GCTB patients in the validation cohort stratified by TSR, postoperative neurological dysfunction, type of resection, Campanacci stage, Ki-67 expression, CD3 +TILs, CD8 +TILs, and PD-L1 +TILs.

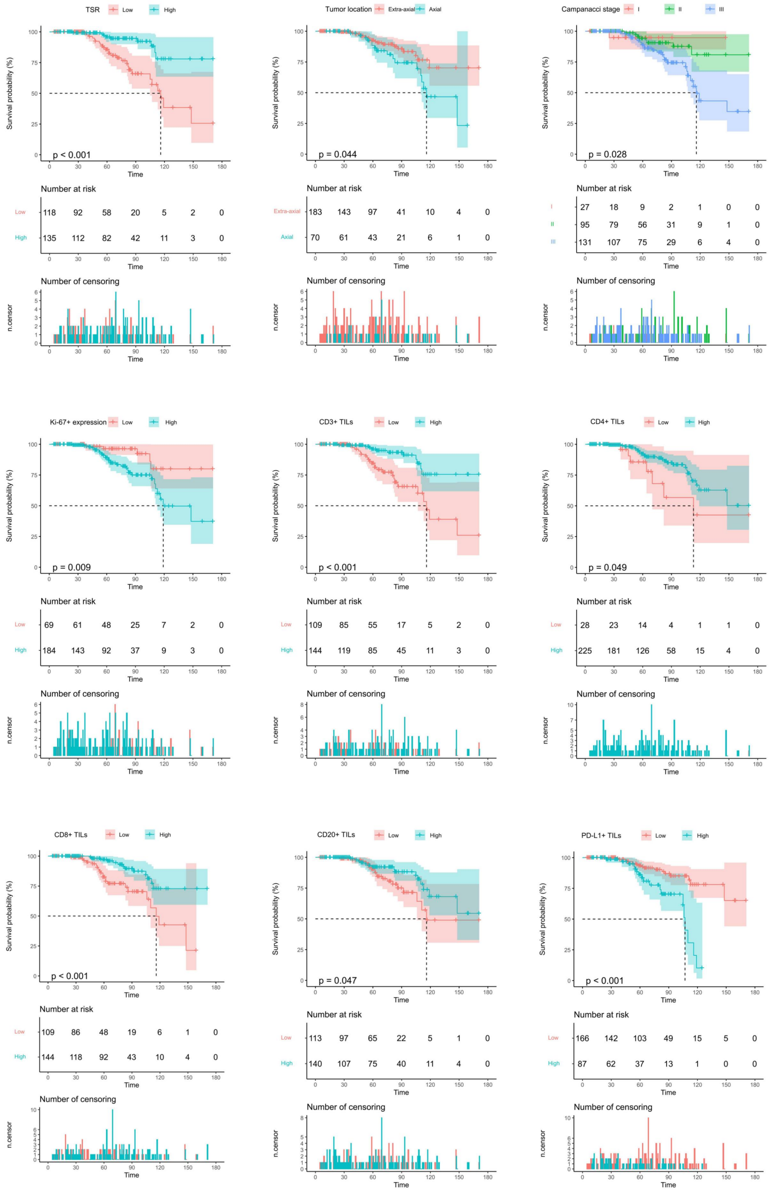

Supplemental Digital Content. Figure 10. Kaplan-Meier curves of overall survival of GCTB patients in the validation cohort stratified by TSR, tumor location, Campanacci stage, Ki-67 expression, CD3 +TILs, CD4 +TILs, CD8 +TILs, CD20 +TILs, and PD-L1 +TILs.

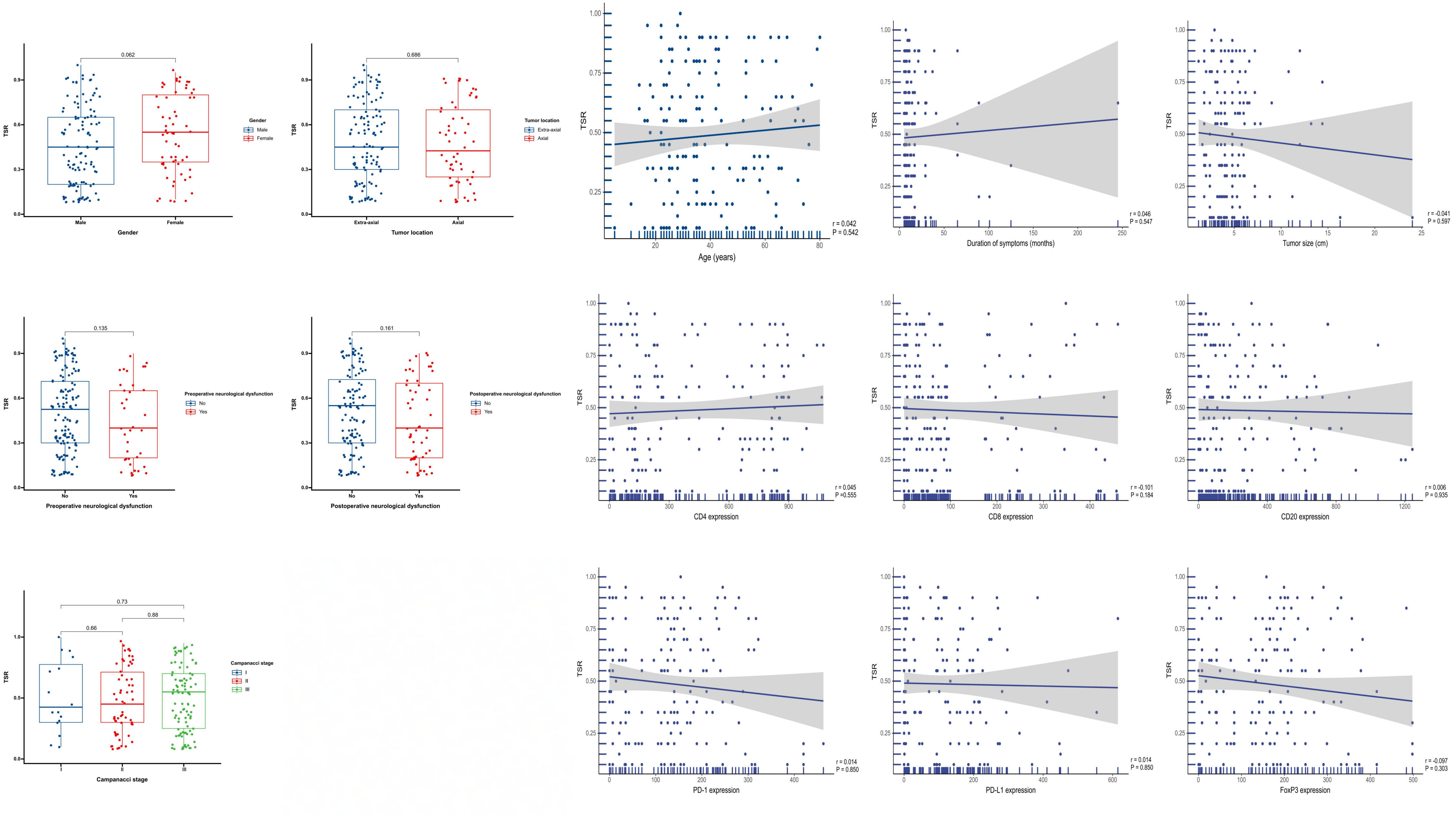

Supplemental Digital Content. Figure 11. Association between TSR and clinicopathological features of GCTB patients in training cohort

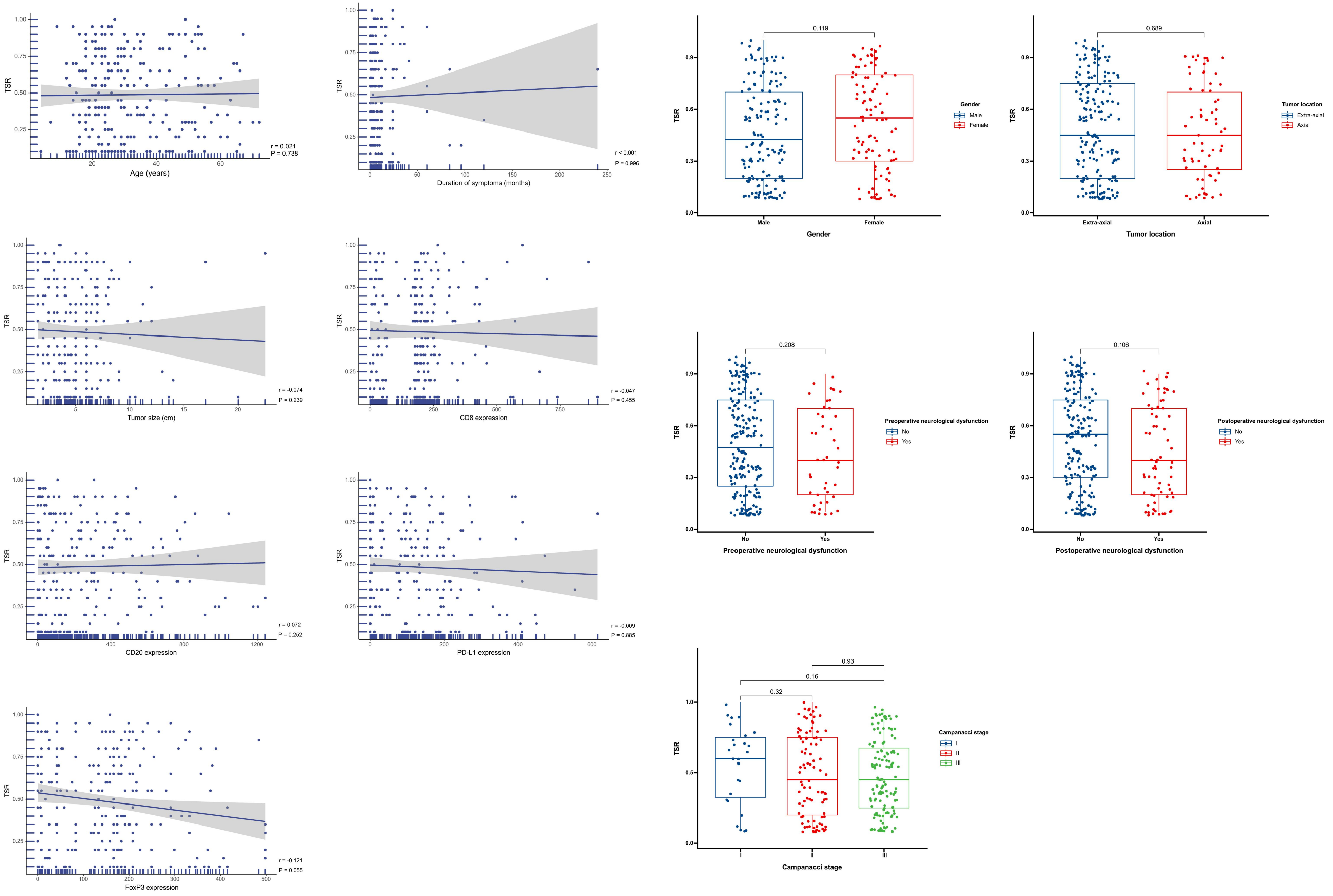

Supplemental Digital Content. Figure 12. Association between TSR and clinicopathological features of GCTB patients in validation cohort
